# Formulation and Evaluation of Fixed-Dose Combination of Bilayer Gastroretentive Matrix Tablet Containing Atorvastatin as Fast-Release and Atenolol as Sustained-Release

**DOI:** 10.1155/2014/396106

**Published:** 2014-01-02

**Authors:** Sanjay Dey, Sankha Chattopadhyay, Bhaskar Mazumder

**Affiliations:** ^1^Department of Pharmaceutical Sciences, Dibrugarh University, Dibrugarh, Assam 786004, India; ^2^Bengal College of Pharmaceutical Sciences and Research, B.R.B. Sarani, Bidhannagar, Durgapur, West Bengal 713212, India; ^3^Radiopharmaceuticals Laboratory, Board of Radiation and Isotope Technology, Variable Energy Cyclotron Centre, Kolkata, West Bengal 700064, India

## Abstract

The objective of the present study was to develop bilayer tablets of atorvastatin and atenolol that are characterized by initial fast-release of atorvastatin in the stomach and comply with the release requirements of sustained-release of atenolol. An amorphous, solvent evaporation inclusion complex of atorvastatin with **β**-cyclodextrin, present in 1 : 3 (drug/cyclodextrin) molar ratio, was employed in the fast-release layer to enhance the dissolution of atorvastatin. Xanthan gum and guar gum were integrated in the sustained-release layer. Bilayer tablets composed of sustained-release layer (10% w/w of xanthan gum and guar gum) and fast-release layer [1 : 3 (drug/cyclodextrin)] showed the desired release profile. The atorvastatin contained in the fast-release layer showed an initial fast-release of more than 60% of its drug content within 2 h, followed by sustained release of the atenolol for a period of 12 h. The pharmacokinetic study illustrated that the fast absorption and increased oral bioavailability of atorvastatin as well as therapeutic concentration of atenolol in blood were made available through adoption of formulation strategy of bilayer tablets. It can be concluded that the bilayer tablets of atorvastatin and atenolol can be successfully employed for the treatment of hypertension and hypercholesterolemia together through oral administration of single tablet.

## 1. Introduction

Hypertension is commonly associated with other cardiovascular risk factors, such as obesity, diabetes, and hypercholesterolemia [1]. Hypercholesterolemia, a strong predictor of cardiovascular disease, causes endothelial damage, and the loss of physiological vasomotor activity that results from endothelial damage may become manifested as increased blood pressure (BP) [2]. Therefore, factors like hypercholesterolemia that cause endothelial dysfunction may lead to hypertension. Borghi et al. have found that patients receiving concomitant antihypertensive and statin therapy experienced a reduction in BP that could not be explained solely by the lipid-lowering effect of the statin or the effect of the antihypertensive medication [3, 4]. These results suggest that the use of statin in combination with antihypertensive drugs may improve BP control in patients, with uncontrolled hypertension and high serum cholesterol levels.

Hypertension and hypercholesterolemia frequently coexist and may require concomitant drug treatment [5]. The combination therapy of atorvastatin and atenolol may be useful and effective in some situations, particularly in serious cardiovascular adverse effects such as severe hypertension, congestive heart failure, and/or exacerbation of angina which may occur along with increasing the cholesterol level in the blood [6], while the fixed-dose combinations (FDCs) remain the preferable choice to the patient as compared to take the single product two times.

Atorvastatin is a member of lipid-lowering agent. It is a potent inhibitor of 3-hydroxy-3-methylglutaryl-coenzyme (HMG-CoA) reductase which catalyzes the conversion of HMG-CoA to mevalonate, an early rate-determining step in cholesterol biosynthesis [7]. It has very good intestinal permeability and short half-life (1-2 h). Added to that, atorvastatin showed a distinct pH-dependent solubility characterized by very poor solubility in aqueous solution of pH 4 and below [8]. It comes under Biopharmaceutical Classification System II (BCS II) category. It is rapidly absorbed at the relevant intestinal pH after oral administration [9, 10]. Its oral bioavailability has been reported 12% due to low aqueous solubility (0.1 mg mL^−1^), crystalline nature, and hepatic first pass metabolism [11]. Poor performance of the drug leads to administration in higher doses possibly leading to liver abnormalities, rhabdomyolysis, arthralgia, and kidney failure.

Atenolol is a cardioselective beta-1 adrenoceptor blocker devoid of intrinsic sympathomimetic and membrane-stabilizing activities [12, 13]. This compound has been widely used for treatment of hypertension, cardiac arrhythmia, and angina [14, 15]. Half-life and oral bioavailability of atenolol have been reported to be 6 to 8 h and 50%, respectively [16, 17]. It has lower absorption window in the lower gastrointestinal tract. Thus, it seems that an increase in gastric residence time may increase the extent of absorption and bioavailability of drug.

The current study was undertaken to formulate bilayer tablets and to modify the release pattern of atorvastatin and atenolol through its incorporation of an oral dosage form that is able to promptly release of atorvastatin in a soluble form in the stomach with the aim of reaching high serum concentration in a short period of time. This action is followed by an extended release of atenolol in the stomach for 12 h to avoid its repetitive administration, improve patient compliance, minimize the incidence of its side effects, and enhance the oral bioavailability of atenolol. To accomplish the goal stated above, bilayer tablets composed of an atenolol sustained-release layer combined with atorvastatin fast-release layer were manufactured by direct compression using conventional tabletting facilities in a simple and easy-to-scale-up formulation strategy.

Cyclodextrins (CDs) are a group of cyclic oligosaccharides, which have been investigated to improve the solubility and dissolution rate of various poorly soluble drugs [18–20]. Moreover, CDs have been successfully employed to modify the release pattern of drugs in several modified-release formulations [21–23]. In addition, it has been reported that the *β*-cyclodextrin (*β*-CD) has been most widely used to improve the oral bioavailability of poorly water-soluble drugs [24–29]. According to the aforementioned reasons, *β*-CD was investigated for its suitability to be included in the fast-release layer of bilayer tablets. Xanthan gum and guar gum were chosen as the candidate matrix-forming material to obtain suitable slow release of the drug from the sustained-release layer present in the prepared bilayer tablets due to biocompatibility, inertness, and its wide application as sustained-release excipients.

In order to reach the goal of this study, solid systems of atorvastatin with *β*-CD were prepared using different techniques, aiming to improve atorvastatin dissolution properties in acidic medium, as a primary step in development of bilayer tablets. The interaction between atorvastatin and *β*-CD was investigated in solution state using phase solubility and in solid state using differential scanning calorimetry (DSC), X-ray powder diffraction (XRD), and infrared (IR) spectroscopy. Preliminary *in vitro* drug dissolution studies for the prepared solid systems were carried out in 0.1 M HCl to choose the solid system with superior dissolution characteristics to be incorporated into the fast-release layer present in the proposed bilayer tablets. Matrix tablet of atenolol containing both xanthan gum and guar gum in concentration of 10% w/w each was initially prepared by direct compression. Subsequently, the *in vitro* release of the tablets was performed to evaluate the capability of the tablets of sustaining atenolol release to be employed in the sustained-release layer of the designed bilayer tablets. Bilayer tablets composed of atorvastatin fast-release layer and atenolol sustained-release layer were prepared by direct compression and their physical properties, *in vitro* release behavior and *in vivo *pharmacokinetic parameters, were evaluated.

## 2. Materials and Methods

### 2.1. Materials

Atenolol and atorvastatin were obtained as gift sample from Cipla Ltd., Mumbai, India. Xanthan gum, guar gum, and *β*-CD were purchased from HiMedia, Mumbai, India. Sodium bicarbonate was purchased from B. D. Pharmaceutical Works, Howrah, India. Citric acid and magnesium stearate were obtained from Loba Chemie Pvt. Ltd., Mumbai, India. Sodium starch glycolate was procured from Mepro Pharma, Vadodara, India. Talc was purchased from Nice Chemie Pvt. Ltd., Mumbai, India. Lactose was purchased from Reidel India Chemicals, Mumbai, India. Sustained-release marketed formulation of atenolol (Aten; manufactured by Zydus Cadila, India) was purchased from local market. Conventional marketed formulation of atorvastatin (Tonact, manufactured by Lupin Ltd., India) was purchased from local market.

### 2.2. Methods

#### 2.2.1. Phase Solubility Studies

The effect of *β*-CD on the solubility of atorvastatin was investigated according to the phase solubility technique reported by Higuchi and Connors [30]. Excess amounts of atorvastatin were added to 20 mL of either double-distilled water or aqueous solutions containing increasing concentrations of CDs, ranging from 5 to 15% w/w, in a series of glass-stoppered vials. The suspensions were shaken at 25 ± 0.5°C for 7 days. Aliquots were withdrawn through a Millipore filter (0.45 *μ*m pore size) and concentrations of atorvastatin were analyzed spectrophotometrically (UV-1700, Shimadzu, Tokyo, Japan) at *λ*_max_ 242 nm. Each experiment was carried out in triplicate.

Phase solubility diagrams were obtained by plotting the molar concentration of solubilised atorvastatin versus the molar concentrations of the CDs used. The apparent stability constants (*K*_*s*_) were estimated from the straight line of the phase solubility diagrams according to the following equation of Higuchi and Connors [30]:

(1) $$\begin{array}{c} K_{s}=\frac{\text{Slope}}{S_{o}\left(1-\text{Slope}\right)}, \end{array}$$

where *S*_*o*_ represents the drug solubility in the absence of CDs (the intercept of the phase solubility diagram).

#### 2.2.2. Preparation of Solid System of Atorvastatin with CDs

Aiming to improve the dissolution behavior of atorvastatin in gastric conditions, solid systems of atorvastatin with *β*-CD were prepared at three molar ratios, namely, 1 : 1, 1 : 2, and 1 : 3 (drug/CD), using the kneading and freeze-drying methods. Physical mixtures were also prepared in the same molar ratios for comparison.

*Physical Mixture*. Physical mixtures of atorvastatin and CDs were prepared by thoroughly mixing the two components in a mortar for 30 min.

*Solvent Evaporation Method*. The calculated amounts of atorvastatin and CD were accurately weighed, transferred to a glass mortar, and triturated with a small volume of ethanol (70% v/v). The slurry obtained was kneaded for 30 min and then dried under vacuum at room temperature in the presence of calcium chloride as a dehydrating agent.

#### 2.2.3. Physicochemical Characterization of Atorvastatin-CD Solid Systems

The DSC thermograms, X-ray diffractograms, and IR spectra were recorded for atorvastatin, CDs, and their solid systems prepared by using different techniques in 1 : 1 (drug/CD) molar ratio.

*Differential Scanning Calorimetry.* The DSC analysis was performed using Diamond differential scanning calorimeter (Perkin Elmer, USA). The apparatus was calibrated with purified indium (99.9%). Samples (3-4 mg) were placed in flat-bottomed aluminum pans and heated at a constant rate 15°C/min in an atmosphere of nitrogen in a temperature range of 20–360°C.

*X-Ray Diffractometry.* The X-ray diffraction patterns were recorded at room temperature using Rigaku Denki diffractometer (MiniFlex 2027, Tokyo, Japan). The samples were irradiated with Ni-filtered CuK*α* radiation at 30 kV voltage and 5 mA current. The scanning rate employed was 4°/min over a diffraction angle of 2*θ* and range of 5–40°.

*Infrared (IR) Spectroscopy.*  The IR spectra were recorded using IR spectrophotometer (Perkin-Elmer, USA) using KBr disc technique. The smoothing of the spectra and the baseline correction procedures were applied. The IR measurements were performed in the scanning range of 4000 to 400 cm^−1^ at ambient temperature.

#### 2.2.4. *In Vitro* Dissolution Studies of Atorvastatin-CD Solid System

Preliminary dissolution tests under gastric conditions, intended for selecting the solid system with superior dissolution properties to be incorporated into the fast-release layer of bilayer tablets, were performed using the U.S. Pharmacopoeia (USP) dissolution apparatus II at 100 rpm [31]. A sample equivalent to 10 mg of atorvastatin was placed in the dissolution vessel containing 900 mL of 0.1 N HCl maintained at 37 ± 0.5°C. At appropriate intervals, samples from the dissolution medium were withdrawn and filtered, and concentrations of atorvastatin were determined spectrophotometrically. The dissolution studies were conducted in triplicate and the mean values were plotted versus time. Additionally, atorvastatin dissolution profiles were evaluated on the basis of the dissolution efficiency parameter at 60 min (DE60, in percent), calculated from the area under the dissolution curves, and expressed as the percent of the area of the rectangle described by 100% dissolution in the same time according to the following equation [32]:

(2) $$\begin{array}{c} \text{DE}=\frac{\int_{0}^{t}y\times dt}{y100\times t}\times 100, \end{array}$$

where *y* is the drug percentage dissolved at time *t*.

#### 2.2.5. Preparation of Bilayer Tablets

In order to prepare bilayer tablets, fast-release atorvastatin and sustained-release atenolol single-layer tablet formulations were initially prepared to gain insight into the dissolution profile of each layer separately with the aim of selecting the best formulations of each that could be combined together to provide bilayer tablets with suitable release pattern characterized by initial fast-release of atorvastatin and sustained-release of atenolol for 12 h in 0.1 M HCl.

*Formulation of the Fast-Release Layer of Atorvastatin.* Table 1 presents the composition of the fast-release tablet formulation of atorvastatin. Atorvastatin-*β*-CD solvent evaporation product in 1 : 3 (drug/CD) molar ratio was selected, based on its superior dissolution properties in 0.1 M HCl, to be incorporated into fast-release layer of atorvastatin of bilayer tablets. Dicalcium phosphate and sodium starch glycolate were added as tablets diluent and superdisintegrant, respectively. They were mixed thoroughly with the atorvastatin-*β*-CD inclusion complex in a glass mortar with the help of pastle for 30 min. Then, talc and magnesium stearate were added as glidant and lubricant, respectively, to the powder blend and mixed for an additional 5 min. The resultant powder blend was compressed under constant pressure using a single-punch tabletting machine (Kilburns, Allahabad, India) into 120 mg tablets, each containing a total of 10 mg ATV. The dissolution behavior of the tablets was examined using the same conditions used for atorvastatin-CD solid system.

*Formulation of the Sustained-Release Layer of Atenolol.* The detailed composition of single-layer sustained-release tablet formulations is presented in Table 2. Each ingredient was sifted through # 80 sieves. The specified quantity (50 mg) of atenolol was mixed with xanthan gum (8.4 mg), guar gum (19.6 mg), sodium bicarbonate (14.0 mg), citric acid (7.0 mg), and spray dried lactose (36.8 mg) in glass mortar with the help of pastle for 30 min. Then, talc (2.8 mg) and magnesium stearate (1.4 mg) were added to the powder blend and mixed for additional 5 min. The powder blend was compressed under constant pressure using a single-punch tabletting machine into 140 mg tablets, each containing 50 mg of atenolol. The dissolution behavior of the tablets was examined using the same conditions used for atorvastatin-CD solid system.

*Formulation of Atorvastatin Fast-Release and Atenolol Sustained-Release Bilayer Tablets.* Tables 1 and 2 provide the detailed composition of atorvastatin fast-release layer and atenolol sustained-release layer for the formulation of bilayer tablets. The bilayer tablets were prepared by direct compression using a single-punch tabletting machine where its die was initially filled with the weighed amount of sustained-release portion and lightly compressed, then the fast-release portion was added directly onto the obtained compressed tablet, and then recompressed together at 7-8 kg cm^2^ to combine them. It was found that, at the compression force of 7-8 kg cm^−2^, there was no layer separation among the two layers of bilayer tablets. The total weight of each bilayer tablet was adjusted to 260 mg, containing 10 mg of atorvastatin in fast-release layer and 50 mg of atenolol in sustained-release layer.

#### 2.2.6. Physical Tests for the Prepared Bilayer Tablets

*Tablet Weight Variation.* The weight variation test of bilayer tablets was performed by randomly selecting twenty bilayer tablets and the average weight of twenty tablets was determined using an electronic balance (Sartorius GmbH, Gottingen, Germany). Then, individual tablets were weighed and the weight of individual tablet was compared with an average weight. The results are expressed as mean values of twenty determinations.

*Drug Content Uniformity.* Ten bilayer tablets were weighed individually and crushed, and the drug was extracted in methanol. The solution was filtered through a Millipore filter (0.45 *μ*m pore size) and the atorvastatin and atenolol contents were determined by *in house* developed validated spectrophotometric method after suitable dilution [33].

*Tablet Friability.* Six tablets were weighed to perform the friability test of bilayer tablets. The tablets were placed in the drum of a tablet friability test apparatus (FAB-2, Logan Instruments Corp., USA). The drum was adjusted to rotate 100 times in 4 min and then the tablets were removed from the drum, dedusted, and accurately weighed. The percent weight loss was calculated.

*In Vitro Buoyancy.* The *in vitro* buoyancy study was performed using USP 24 type II apparatus (Timestan, Kolkata, India) at 100 rpm in 0.1 M HCl (900 mL). The temperature of the medium was maintained at 37 ± 0.5°C. The time required for tablet to rise to the surface of the medium and duration of time the tablet constantly float on medium were noted as floating lag time and total floating time, respectively [32].

#### 2.2.7. *In Vitro* Drugs Release of Bilayer Tablets

*In vitro* drugs release studies of the bilayer tablets were performed using the same method used for atorvastatin-CD solid system. We have performed the release study at 100 rpm to provide as much as hazardous condition to evaluate whether the polymers of sustained-release layer are capable of sustained-release of atenolol from sustained-release layer or not. The drugs content in the samples were determined by applying the simultaneous equation method which was developed and validated in our laboratory [33].

#### 2.2.8. Pharmacokinetic Study

The pharmacokinetic studies were conducted under approval of the Institutional Animal Ethical Committee of Gayatri College of Pharmacy, Odisha, India. For the experiment, healthy rabbits (New Zealand albino) of either sex weighing 2.5–3.0 Kg were acclimatized in the animal house for 15 days and fasted for 12 h prior to dose administration with free access to drinking water. The pharmacokinetic study of conventional marketed formulation of atorvastatin (Tonact), sustained-release marketed formulation of atenolol (Aten), and bilayer tablets was carried out with the following study design: single dose, open label, two periods, two treatments, randomized, and complete crossover design under fasted condition. Washout period of one week was allowed between successive runs.

Three tablets of each formulation were administered orally to three rabbits of each group along with 10 mL of water by using a feeding tube. The blood samples (each of about 2 mL from each animal) were withdrawn from orbital sinus with a 24-G, 1-in. needle and collected directly in tubes containing 300 *μ*L of sodium citrate solution (2%, w/v) as anticoagulant. Blood samples were collected at 0 (pretreatment), 0.5, 1, 3, 6, 8, 12, 24, and 36 h.

The collected blood samples were immediately centrifuged at 4,000 ×g for 10 min at 4°C. The supernatant plasma sample was separated and stored in a clean screw capped 5 mL polypropylene plasma tubes (Laxbro, Mumbai, India) at −20°C in a deep freezer, until further analysis.

*Plasma Sample Analysis.* Hundred microliter of plasma sample was taken in a 2 mL glass centrifuge tube and 10 *μ*L of diltiazem solution (50 *μ*g/mL) was added as an internal standard (I.S.). The mixture was vortexed for 10 seconds. Acetonitrile (1.5 mL) was added into the mixture, vortexed for 10 min using a sphinix vortexer (M37610-33, Barnstead International, USA), and centrifuged (Biofuge Fresco centrifuge, Heraeus, Germany) for 5 min at 10,000 rpm. The organic layer was aspirated off and transferred to a second tube by means of disposable Pasteur pipette. The collected organic layer was evaporated to dryness under nitrogen gas flow using nitrogen gas evaporator (Glas-COL, USA) at 25 psi at 40°C. The residue was reconstituted with 100 *μ*L of reconstitution solvent and the solution was vortexed for 1 min using multipulse vortexer (Glas-COL, USA). The samples were filtered through membrane filter (0.45 *μ*m) using syringe filter. An aliquot of 20 *μ*L was injected into the injector of the HPLC system (Hypersil BDS C_18_ column, 250 × 4.6 mm I.D., 5 *μ*m particle size, 1 mL/min flow rate, retention time of atenolol, I.S., and atorvastatin are 8.75 ± 0.04 min, 10.93 ± 0.02 min, and 12.10 ± 0.05 min). The area under the curve of peaks of atenolol, atorvastatin, and I.S. was determined and the concentration of drug present in sample was estimated by using the linear regression equation of standard calibration curves (concentration of atenolol versus ratio of atenolol to I.S. and concentration of atorvastatin versus ratio of atorvastatin to I.S.).

*Determination of Pharmacokinetic Parameters*. The pharmacokinetic parameters were determined from the data of plasma drug concentration at different time points by using MS-Excel 2007 Software according to the procedure described elsewhere [34, 35].

#### 2.2.9. Stability Study

Stability study of bilayer tablets was performed as per ICH guideline. The bilayer tablets were kept in polypropylene bottle and stored in stability chambers maintained at 40 ± 2°C and 75 ± 5% RH for six months. Samples were checked initially after three months and further after six months.

## 3. Results and Discussion

### 3.1. Phase Solubility Studies

**Figure 1:**
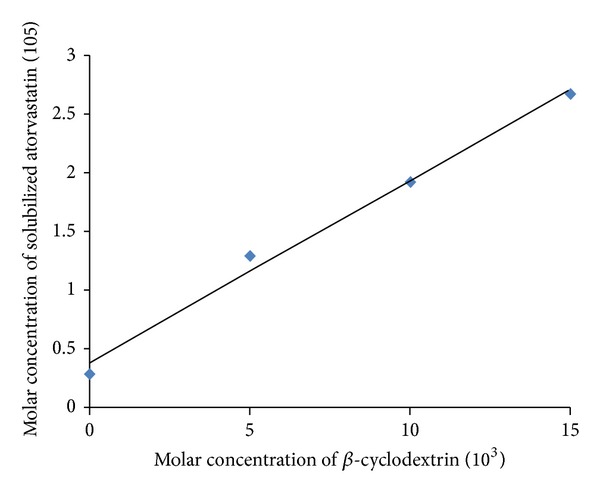
Phase solubility diagram of atorvastatin with *β*-cyclodextrin in distilled water at 25 ± 0.5°C.

The phase solubility diagrams of atorvastatin with *β*-CD in distilled water performed at 25 ± 0.5°C are shown in Figure 1. It is apparent that the solubility of atorvastatin increased as the concentrations of CD increased. The values of stability constant (*K*_*s*_) calculated from the equation of Higuchi and Connors [30] were found to be 23.52 ± 1.45 M^−1^. The coefficient of determination (*r* ^2^) value of the phase solubility diagram was <0.990; therefore, this diagram was classified as A_L_-type phase diagram [36]. Such positive deviation from linearity suggests the formation of higher-order inclusion complexes between atorvastatin and CD attributed to the formation of complex aggregates that could solubilize additional amount of the guest molecules through noninclusion complexation or formation of micelle-like structure [37]. The calculated higher value of *K*_*s*_ indicates that atorvastatin interacts more strongly with *β*-CD and this may be attributed to the better wetting ability and greater solubilizing power of *β*-CD towards atorvastatin. Similar results were observed in the literature for valdecoxib [38], tadalafil [18], and efavirenz [19] when solubilized with *β*-CD.

### 3.2. Physicochemical Characterization of Atorvastatin-CD Solid System

**Figure 2:**
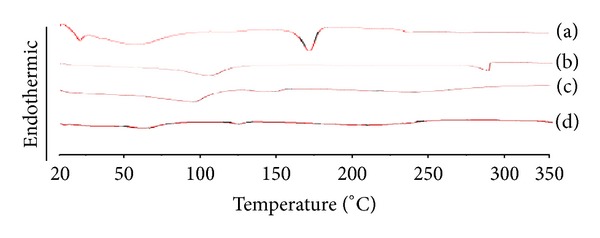
DSC thermograms of atorvastatin-*β*-CD solid systems in 1 : 2 (drug/CD) molar ratio: (a) atorvastatin powder; (b) pure *β*-CD; (c) physical mixture of atorvastatin with *β*-CD; (d) solvent evaporation product of atorvastatin with *β*-CD.

*Differential Scanning Calorimetry.* DSC thermogram of atorvastatin, CD, and their solid systems prepared by different techniques at a molar ratio of  1 : 2 (drug/CD) is presented in Figure 2. The DSC thermogram of atorvastatin was typical to a crystalline substance, exhibiting a sharp endothermic peak at 160.2°C corresponding to its melting and decomposition [39]. The thermogram of the physical mixture of atorvastatin with *β*-CD showed the existence of the drug endothermic peak which could indicate the absence of interaction between atorvastatin and CD. However, a marked reduction in atorvastatin peak intensity was observed in the aforementioned systems and could be low drug to CD molar ratio (1 : 2). On the other hand, the drug-melting endotherm was recorded in the solvent evaporation system but with noticeable broadening and reduction in intensity which could be ascribed to increase in the drug-CD interaction as a consequence of the more drastic mechanical treatment during solvent evaporation compared to physical mixing. There is shifting of the atorvastatin endotherm in inclusion complex which might be due to lowering the melting point of drug and *β*-CD as they are present in a mixture. This could indicate drug amorphization and/or its interaction with the carrier caused by the supply of thermal energy during the DSC scan process [40]. Therefore, X-ray powder diffractometry was considered in conjunction with DSC analysis to reach a definite conclusion.

**Figure 3:**
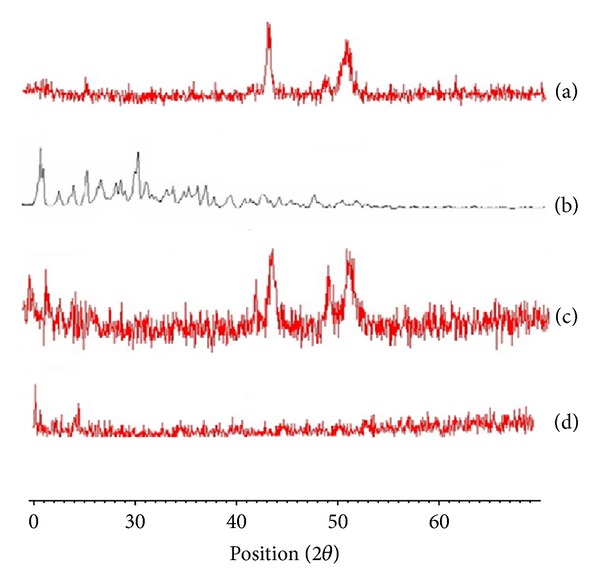
X-ray diffraction patterns of atorvastatin-*β*-CD solid systems in 1 : 2 (drug/CD) molar ratio: (a) atorvastatin powder; (b) pure *β*-CD; (c) physical mixture of atorvastatin with *β*-CD; (d) solvent evaporation product of atorvastatin with *β*-CD.

*X-Ray Powder Diffractometry.* The XRD patterns for individual components and their solid systems prepared by different techniques at 1 : 2 (drug/CD) molar ratio are presented in Figure 3. The diffraction pattern of atorvastatin powder revealed several sharp high intensity peaks at diffraction angles 2*θ* of 9.41°, 10.18°, 10.51°, 11.9°, and 19.12°, suggesting that it existed as a crystalline material. *Β*-CD showed a crystalline diffractogram. Generally, the diffraction patterns of the investigated physical mixtures correspond to the superposition of those of the individual components and revealed that atorvastatin was present in a crystalline state, as evidenced by its diffraction lines, and thereby ruled out the existence of drug-carrier interaction in these physical mixtures. However, slight decrease in atorvastatin crystalline character was observed in the diffractograms of the solvent evaporation system evidenced by the noticeable decrease in the number and intensities of peaks present in their X-ray diffractogram when compared to the corresponding physical mixture. This finding might be attributed to the reduction in the drug particle size during the solvent evaporation process and/or the presence of an interaction between the drug and CD during the drying process [41].

**Figure 4:**
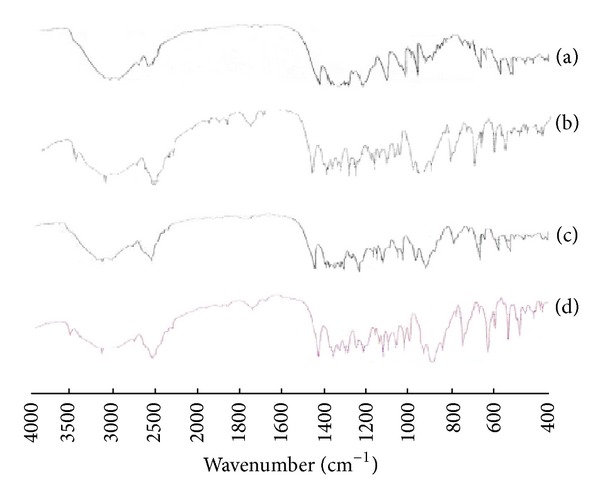
IR spectra of atorvastatin-*β*-CD solid systems in 1 : 2 (drug/CD) molar ratio: (a) atorvastatin powder; (b) pure *β*-CD; (c) physical mixture of atorvastatin with *β*-CD; (d) solvent evaporation product of atorvastatin with *β*-CD.

*IR Spectroscopy.* The IR spectra of individual components and their solid systems prepared by different techniques at 1 : 2 (drug/CD) molar ratio are presented in Figure 4. The IR spectrum of atorvastatin showed characteristic peaks at 3,365 cm^−1^ and 3,065 cm^−1^ corresponding to –OH stretching vibration and C–HO stretching vibration of alcoholic group, respectively. Intense absorption peak was found at 1,557 cm^−1^ due to the stretching vibration of the C=O group in primary amide. Other peaks were observed at 1,650 and 1,105 cm^−1^ and were assigned to bending vibrations of the C=C and O–H, respectively. The stretching vibrations of C–N, C–F, and C–O groups appeared at 1,315, 1,220, and 1,109 cm^−1^. The IR spectrum of the investigated CD illustrated intense broad absorption bands at 3,400–3,100 cm^−1^ corresponding to the free –OH stretching vibrations. The vibration of the –CH and –CH_2_ groups appeared in the region 2,950–2,600 cm^−1^. A shorter band appeared in the region 1,500–1,200 cm^−1^ that could be ascribed to the hydrated bonds within CD molecules. Another large band assigned to the C–O–C stretching vibration occurred between 1,200 and 1,030 cm^−1^. The two intense peaks due to carbonyl stretching of atorvastatin at 1,557 cm^−1^ and the C=C bending present at 1.650 cm^−1^ were the main characteristics bands used to access the drug-CD interactions due to absence of overlapping between those peaks and CD peaks.

The IR spectra of the investigated physical mixtures did not show any significant shift with respect to the IR spectra of the components and, in particular, the characteristic carbonyl stretching and the N–H bending of atorvastatin. However, the same band was diminished in the spectrum of its solvent evaporation product when compared to the corresponding physical mixture, suggesting partial interaction of the drug with the *β*-CD molecule. This might indicate the inclusion of atorvastatin in the hydrophobic cavity of the carrier [18, 38]. Taking into account the above results together with those obtained from the DSC and X-ray studies, they all supported an almost complete transformation of the crystalline drug to an amorphous state and the existence of strong interaction between the drug and *β*-CD when the solvent evaporation method was used.

### 3.3. *In Vitro* Dissolution Studies for Atorvastatin-CD Solid Systems

**Figure 5:**
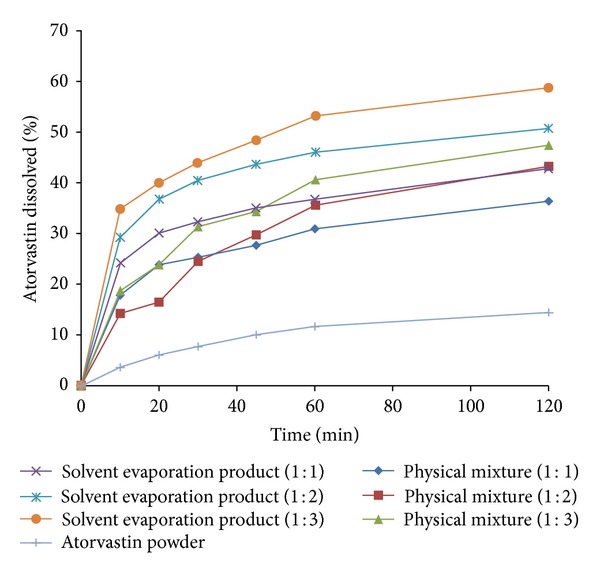
Dissolution profile of atorvastatin from physical mixtures and solvent evaporation products containing atorvastatin and *β*-CD in 1 : 1, 1 : 2, and 1 : 3 (drug/CD) molar ratios in comparison to atorvastatin powder performed in 0.1 M HCl at 37 ± 0.5°C.

Figure 5 illustrates the dissolution profiles of the atorvastatin-CD solid systems in 0.1 M HCl and Table 3 represents a compilation of their dissolution efficiency data calculated based on 60 min (DE_60_). Actually, atorvastatin dissolved very slowly under the specified dissolution conditions and less than 10% of atorvastatin was dissolved after 2 h. Generally, atorvastatin dissolution was improved from all of the investigated solid systems and this improvement depended on the preparation method and drug to CD molar ratio.

It is quite evident that the preparation method affected atorvastatin dissolution from the investigated solid systems [18]. Atorvastatin dissolution was enhanced when physically mixed with CD due to local solubilization action of the carrier operating in the aqueous microenvironment surrounding the drug [42]. The solvent evaporation product showed marked increment in atorvastatin dissolution compared to the corresponding physical mixtures probably due to the increase in drug-carrier contact surface as a consequence of the more drastic mechanical treatment during the preparation and/or the formation of soluble inclusion complexes of the drug with CD accompanied by reduction of its crystallinity following complexation as reported in the literature [18, 19].

In addition, it was obvious that atorvastatin dissolution was enhanced on increasing the molar ratio of CD in the investigated solid systems. Physical mixtures showed the least effect for the molar ratio since the observed enhancement in dissolution is mainly due to the wetting effect of the CD [42]. Conversely, the most pronounced effect for the molar ratio was observed for the solvent evaporation products due to better dispersion and/or inclusion of the drug with increasing CD molar ratio during preparation.

The results of the two-way ANOVA performed on the DE_60_ data revealed the presence of significant differences among the preparation methods and molar ratios at *P* ≤ 0.05. The computed *F* values indicated that the dissolution of atorvastatin from its solid systems depended mostly on the preparation method followed by the molar ratio. Multiple comparisons between the different methods at each molar ratio according to Scheffé's test revealed that the solvent evaporation technique exhibited the most significant effect on the dissolution enhancement of atorvastatin compared to the other methods at *P* ≤ 0.05. In addition, multiple comparisons between the three molar ratios employed in the solvent evaporation product of each CD according to Scheffé's test revealed that the molar ratio of 1 : 3 (drug/CD) exhibited the most significant improvement on the dissolution efficiency compared to the other molar ratio at *P* ≤ 0.05. These results confirmed that the solvent evaporation systems prepared at 1 : 3 (drug/CD) molar ratio showed the most superior and significant enhancement effect on the dissolution pattern of atorvastatin. Therefore, the solvent evaporation system atorvastatin-*β*-CD, prepared at 1 : 3 (drug/CD) molar ratio, was chosen for further incorporation into the tablet formulations.

### 3.4. *In Vitro* Drug Release Studies for the Fast-Release and Sustained-Release Tablets

**Figure 6:**
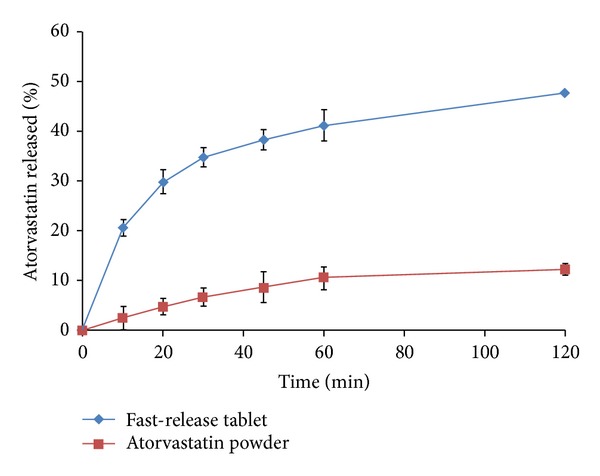
*In vitro* release profile of atorvastatin from fast-release tablets containing atorvastatin-*β*-CD solvent evaporation product in 1 : 3 (drug/CD) molar ratio in comparison to atorvastatin powder performed in 0.1 M HCl at 37 ± 0.5°C (mean ± S.E.; *n* = 6).

Figure 6 presents the dissolution profile of atorvastatin from the prepared fast-release tablets in comparison to the dissolution profile of atorvastatin in 0.1 M HCl. It is worth noting that sodium starch glycolate was used as a superdisintegrant in these tablet formulations to cause their immediate disintegration when exposed to the dissolution media, thus enhancing rapid release of the drug [43]. It is quite clear that the prepared fast-release tablet formulation, containing atorvastatin-*β*-CD solvent evaporation complex in 1 : 3 (drug/CD) molar ratio, has shown manifested improvement in drug dissolution properties in acidic conditions when compared to atorvastatin. This can be ascribed to the decrease in crystallinity of atorvastatin as a result of its complexation with *β*-CD during solvent evaporation process as evidenced from its physicochemical characterization described previously.

**Figure 7:**
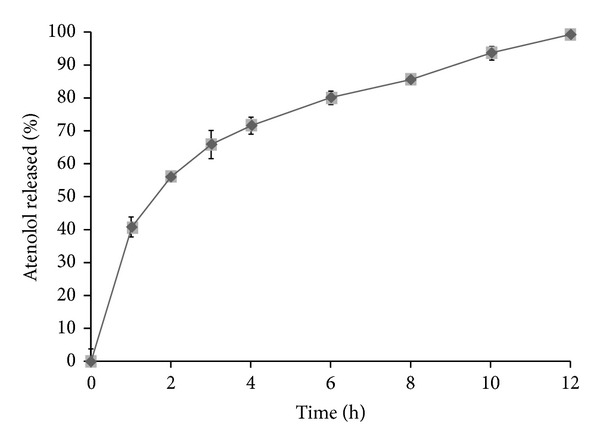
*In vitro* release profile of atenolol from sustained-release tablet performed in 0.1 M HCl at 37 ± 0.5°C (mean ± S.E.; *n* = 6).

Figure 7 illustrates the *in vitro* release profiles of atenolol from the prepared sustained-release tablets containing xanthan gum and guar gum. To simulate the conditions that exist in human GI stomach as the tablet retain in the stomach, the release studies were performed in 0.1 M HCl for 12 h. The release profile showed sustained-release of drug in acidic environment. However, complete drug dissolution was displayed at 12 h. Tablets containing 10% w/w of xanthan gum and 10% w/w of guar gum of total tablet weight were able to keep their integrity and showed a good control on atenolol release. The extent of atenolol release from these tablets, after 12 h dissolution period, was 98.63%. The slower release of drug might be due to the formation of a highly viscous thick gel layer, on the surface of the tablets, characterized by slower erosion rate of polymers. Therefore, in view of the above-mentioned results, formulation containing 10% w/w of xanthan gum and 10% w/w of guar gum of total tablet weight was able to sustain atenolol release and was selected for preparing the sustained-release layers present in the proposed bilayer tablets.

### 3.5. Preparation and Physical Characterization of Atorvastatin Fast-Release and Atenolol Sustained-Release Bilayer Tablets

Tables 1 and 2 represent the compositions of the prepared bilayer tablets containing atorvastatin fast-release layer and atenolol sustained-release layer, respectively. In light of the previous results presented in our study, the fast-release layer contained the solvent evaporation inclusion complex of atorvastatin with *β*-CD in 1 : 3 (drug/CD) molar ratio to attain an initial rapid release of atorvastatin in the stomach. However, the sustained-release layer contained atenolol embedded in hydrophilic natural polymers containing xanthan gum and guar gum in concentration of each 10% w/w of total weight of sustained-release layer.

The comparison of the physical properties of the prepared bilayer tablet formulation is shown in Table 4. The average weight of the formulation was found to be 258.20 ± 2.04 mg. The percentage of weight variation of individual tablets from the average weight was found to be within ±5% (w/w) which proved that the bilayer tablet has passed the USP weight variation test. The results indicated that bilayer tablets have passed the USP criteria for the drug content of tablets. It was observed that the bilayer tablets had passed the USP criteria of friability testing (<1%, w/w). The hardness of the bilayer tablet was found to be 7.30 ± 0.12 kg cm^−2^. The drug content of the bilayer tablets was found to be 98.52 ± 2.10% of atorvastatin and 98.62 ± 1.63% of atenolol. The floating lag time and floating time were found to be 8.02 ± 0.42 min and 12.16 ± 0.57 h, respectively.

### 3.6. *In Vitro* Drug Release Studies for Bilayer Tablets

**Figure 8:**
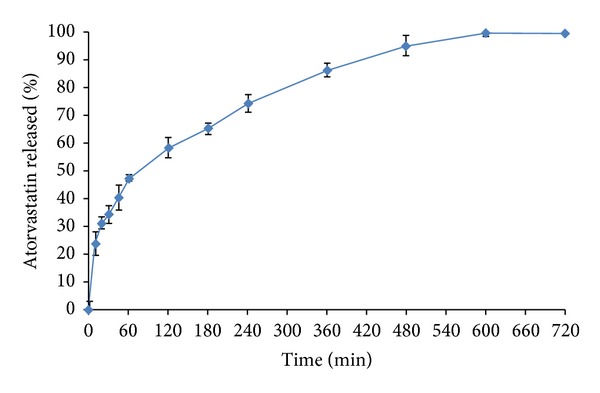
*In vitro* release profile of atorvastatin from the prepared bilayer tablets performed in 0.1 M HCl of pH 1.2 at 37 ± 0.5°C (mean ± S.E.; *n* = 6).

**Figure 9:**
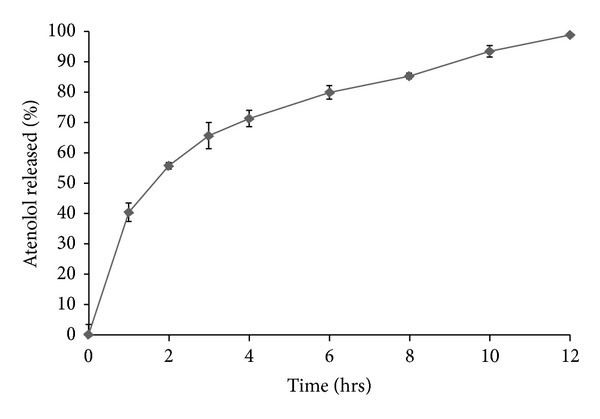
*In vitro* release profile of atenolol from the prepared bilayer tablets performed in 0.1 M HCl of pH 1.2 at 37 ± 0.5°C (mean ± S.E.; *n* = 6).

Figures 8 and 9 present the release profiles of atorvastatin and atenolol, respectively, from the prepared bilayer tablets. The bilayer tablets showed fast-release of more than 60% of atorvastatin in 0.1 M HCl during 2 h of the release study. This was attributed to the prompt disintegration of the fast-release layer, followed by the rapid dissolution of the incorporated atorvastatin-*β*-CD solvent evaporation inclusion complex. It is evident that the sustained-release layer of bilayer tablets showed the sustained release of atenolol. The drug release profile parameters for sustained-release products were calculated as per Robinson Erikson equation [44]: after 1 h, 30–35% of the atenolol is released; after 6 h, 60–65% of atenolol is released; and finally, after 12 h, remaining drug is released. For assessment and comparison to these release specifications, the percent of atenolol released from the prepared bilayer tablets after 1, 6, and 12 h was extracted directly from the release data and was graphically presented in Figure 9. It is evident that the sustained-release layer of bilayer tablets containing 10% w/w of xanthan gum and 10% w/w of guar gum of total weight of sustained-release layer exhibited release profile that fulfilled the above-mentioned release requirements; the sustained-release layer released approximately 33.58%, 64.04%, and remaining 36.96% of atenolol at 1, 6, and 12 h, respectively. As can be seen, these bilayer tablets also illustrated a fast-release of atorvastatin more than 60% during the first 2 h of the release study, so they are expected to overcome the disadvantages associated with the delayed dissolution of atorvastatin in acidic conditions.

### 3.7. Pharmacokinetic Study

**Figure 10:**
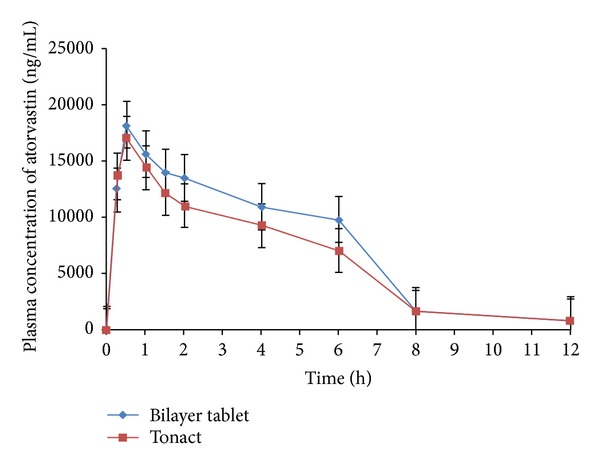
Mean plasma concentration versus time (mean ± S.E.) profile of atorvastatin following oral administration of marketed conventional formulation (Tonact) and bilayer tablets in healthy rabbits (*n* = 3).

**Figure 11:**
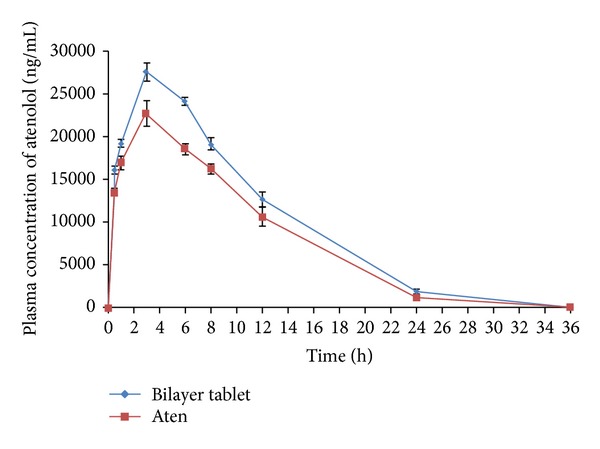
Mean plasma concentration versus time (mean ± S.E.) profile of atenolol following oral administration of marketed ATL sustained release formulation (Aten) and bilayer tablets in healthy rabbits (*n* = 3).

The results of plasma atorvastatin concentration at different time intervals, after administration of Tonact and bilayer tablets, are presented in Figure 10. The mean plasma atenolol concentration at different time intervals, after administration of Aten and bilayer tablets, is presented in Figure 11. The comparison of the different parameters was done by using a one-way analysis of variance (ANOVA). A value of *P* < 0.05 was considered statistically significant.

The pharmacokinetic parameters were derived from plasma atorvastatin concentration versus time profile of all the subjects and the results are shown in Table 5. The average peak plasma concentration (*C*_max_) of atorvastatin from bilayer tablets was found to be 17.00 ± 4.47 *μ*g mL^−1^, which was lower than that of the bilayer tablets (18.19 ± 5.10 *μ*g mL^−1^). There was 1.07-fold increased in *C*_max_ of atorvastatin from bilayer tablets which was observed as compared to Tonact. The time required to reach maximum plasma concentration (*t*_max_) of both of the formulations was found to be approximately similar (0.52 ± 0.34 h for Tonact and 0.54 ± 0.29 h for bilayer tablet), which indicates that rate of absorption from both of the formulations was identical. The AUC_0-*∞*_ of Tonact and bilayer tablets was found to be 81.04 ± 22.29 *μ*g h mL^−1^ and 106.47 ± 31.48 *μ*g h mL^−1^, respectively. The statistical insignificant difference (*P* > 0.05) among the different pharmacokinetic parameters of bilayer tablets and Tonac was observed. The AUC_0-*∞*_ was found to 1.09 times increase in bilayer tablets than that of Tonact. The pharmacokinetic analysis of the plasma level data confirmed that the oral bioavailability of atorvastatin was enhanced upon oral administration of bilayer tablets containing inclusion complex than Tonact in rabbit stomach. The increase in oral bioavailability might be attributed to the increase in solubility, enhancement of dissolution rate, and conversion of crystalline from of ATV to the amorphous state during preparation of inclusion complex by solvent evaporation method. Liu and Desai also reported the use of *β*-CDs in oral formulations which increased rate and extent of oral bioavailability of rofecoxib [45]. There are several reports that have been shown that the aqueous solubility and dissolution rate of poorly soluble drugs were significantly increased *in vitro* by cyclodextrin complexation [46–48]. The AUMC_0–*∞*_ of the bilayer tablets was found to be higher 780.37 ± 102.84 *μ*g h^2^ mL^−1^ as compared to low value of Tonact (334.89 ± 79.35 *μ*g h^2^ mL^−1^). The *K*_el_ value for bilayer tablet was found to be 0.21 ± 0.03 h^−1^, which was similar to the *K*_el_ value obtained in Tonact (0.23 ± 0.01 h^−1^). The pharmacokinetic parameters depicted the significant improvement in oral bioavailability of atorvastatin by oral administration of its inclusion complex with *β*-CD in rabbits, owing to faster *t*_max_ and higher *C*_max_.

The pharmacokinetic parameters were derived from plasma atenolol concentration versus time profile of the bilayer tablets and Aten is presented in Table 5. The *t*_max_ of both the bilayer tablets (3.08 ± 1.89 h) and Aten (3.11 ± 2.21 h) was found to be similar which indicated the slow absorption rate of atenolol from both of the formulations. The average *C*_max_ value of bilayer tablets was decreased as compared to Aten (26.55 ± 8.24 *μ*g mL^−1^ to 27.53 ± 3.53 *μ*g mL^−1^). The AUC_0-∞_ of bilayer tablets exhibited high value (347.12 ± 74.67 *μ*g h mL^−1^) as compared to Aten (342.65 ± 62.82 *μ*g h mL^−1^). The AUMC_0–*∞*_ of bilayer tablets was found to be higher (2847.31 ± 96.49 *μ*g h^2^ mL^−1^) as compared to the low value of Aten (2796.51 ± 52.50 *μ*g h^2^ mL^−1^). The *K*_el_ value for bilayer tablets and Aten was found to be 0.07 ± 0.01 h and 0.19 ± 0.01 h, respectively. The mean residence time (MRT) of bilayer tablet was found to be higher (8.20 ± 0.94 h) than that of Aten (8.16 ± 0.15 h). The statistical insignificant (*P* > 0.05) difference between the different parameters of bilayer tablets and Aten was observed. The results revealed that the atenolol was made available in the body in a controlled release manner. The pharmacokinetic parameters also demonstrated that the controlled release of atenolol from the bilayer tablets not only delayed to reach peak plasma concentration but also prolonged the plasma concentration of atenolol upto 12 h. As can be seen, these bilayer tablets illustrated a fast absorption of atorvastatin and increased oral bioavailability of atorvastatin and the atenolol was made available to the body in a controlled manner for prolonged period of time. The pharmacokinetic results revealed that the bilayer tablets not only were a suitable combination for the treatment of hypertension and hypercholesterolemia through administration of single dose unit but also increased the bioavailability of both of the drugs as compared to marketed formulations.

### 3.8. Stability Study

The bilayer tablet was evaluated for various parameters (drug content and dissolution study) after 3 and 6 months of storage at accelerated stability conditions (40 ± 2°C and 75 ± 5% RH). There were no significant changes in the amount of both of the drugs which were observed in tablets after 6 months of storage at accelerated stability conditions. The dissolution profile of formulation at initial stage was considered as the reference for dissolution study. The results obtained revealed that the dissolution profile of the formulation after 6 months of storage at accelerated condition was found to be similar to that of reference one. Based on the results, it was opinioned that the bilayer tablet was stable after 6 months of storage at accelerated stability conditions. The results also revealed that the bilayer tablet might provide a minimum shelf life of 2 years.

## 4. Conclusion

In the present study, the proposed bilayer tablets were confirmed to be a successful tool for providing the fast-release of atorvastatin and the desired sustained-release of atenolol for prolonged period of time up to 12 h. These tablets were composed of sustained-release layer and prepared using 10% w/w of xanthan gum and 10% w/w of guar gum of total weight of sustained-release layer and fast-release layer, containing atorvastatin-*β*-CD solvent evaporation product in 1 : 3 (drug/CD) molar ratio which was proven to be advantageous in the context of enhancing atorvastatin dissolution characteristics in acidic medium. Bilayer tablets showed acceptable physical properties and elicited the required *in vitro* release pattern that coincides with the purpose set for this study. The pharmacokinetic study illustrated the fast absorption of atorvastatin, increased oral bioavailability of atorvastatin, and maintained the therapeutic concentration of atenolol in blood which was made available through adoption of formulation strategy of bilayer tablets. Further, *in vivo* pharmacodynamic study is required to assess the effectiveness of the proposed bilayer tablet formulation for the treatment of hypertension and hypercholesterolemia together through oral administration of single tablet.

## Figures and Tables

**Table 1:** Composition of atorvastatin fast-release tablet formulation.

Ingredient	Amount (mg)
Atorvastatin-*β*-CD solvent evaporation product in 1 : 3 (drug/CD) molar ratio	40.00
Sodium starch glycolate	5.00
Magnesium stearate	1.08
Talc	5.40
Dicalcium phosphate	q.s.
Total weight	120.00

q.s.: quantity sufficient.

**Table 2:** Composition of atenolol sustained-release tablet formulation.

Ingredient (mg)	Amount (mg)
Atenolol	50.0
Xanthan gum	14.0
Guar gum	14.0
Sodium bicarbonate	14.0
Citric acid	7.0
Talc	2.8
Magnesium stearate	1.4
Spray dried lactose	q.s.
Tablet weight	140.0

q.s.: quantity sufficient.

**Table 3:** Dissolution efficiency of atorvastatin from solid system with *β*-CD prepared using different preparation techniques.

Solid system	Dissolution efficiency (DE_60_, %)^a^
Atorvastatin-*β*-CD (1 : 1) physical mixture	56.80 ± 2.31
Atorvastatin-*β*-CD (1 : 2) physical mixture	61.71 ± 4.28
Atorvastatin-*β*-CD (1 : 3) physical mixture	71.20 ± 3.39
Atorvastatin-*β*-CD (1 : 1) solvent evaporation product	69.01 ± 3.03
Atorvastatin-*β*-CD (1 : 2) solvent evaporation product	84.51 ± 1.56
Atorvastatin-*β*-CD (1 : 3) solvent evaporation product	96.42 ± 2.69

^a^Calculated from the area under the dissolution curve after 60 min.

**Table 4:** Physical characteristics of bilayer tablets formulation.

Parameter	Experimental value	
Drug content (*n* = 3)	Atorvastatin	98.52 ± 2.10%
	Atenolol	98.62 ± 1.63%
Hardness (*n* = 6)	7.30 ± 0.12 kg cm^−2^	
Friability (*n* = 6)	0.54 ± 0.12%	
Weight variation (*n* = 20)	258.2 ± 2.04 mg	
Floating lag time (*n* = 6)	8.02 ± 0.42 min	
Floating duration (*n* = 6)	12.16 ± 0.57 h	

**Table 5:** Pharmacokinetic parameters for Tonact, Aten, and bilayer tablets (containing 10 mg of atorvastatin and 50 mg of atenolol) after oral administration to healthy rabbit (*n* = 3).

Parameter^a^	Tablet			
	Tonact	Aten	Bilayer tablet	
			Atorvastatin	Atenolol
*C*_max_ (*µ*g mL^−1^)	17.00 ± 4.47	27.53 ± 3.53	18.19 ± 5.10	26.55 ± 8.24
*T*_max_ (h)	0.52 ± 0.34	3.11 ± 2.21	0.54 ± 0.29	3.08 ± 1.89
AUC_0–*∞*_ (*µ*g h mL^−1^)	81.04 ± 22.29	342.65 ± 62.82	106.47 ± 31.48	347.12 ± 74.67
AUMC_0–*∞*_ (*µ*g h^2^ mL^−1^)	334.89 ± 79.35	2796.51 ± 52.50	780.37 ± 102.84	2847.31 ± 96.49
MRT (h)	5.29 ± 0.48	8.16 ± 0.15	7.33 ± 0.62	8.20 ± 0.94
*t*_1/2_ (h)	3.04 ± 0.41	7.54 ± 0.64	3.31 ± 0.24	9.90 ± 0.55
*K*_el_ (h^−1^)	0.23 ± 0.01	0.10 ± 0.01	0.21 ± 0.03	0.07 ± 0.01

^a^ *T*_max_: time to reach plasma concentration (*C*_max_); AUC: area under plasma concentration-time curve; AUMC: area under the first momentum plasma concentration-time curve; MRT: mean residence time; *t*_1/2_: plasma half-life of drug; *K*_el_: elimination rate constant.
